# Supplementary material for: The E. coli DnaX clamp loader sharply bends DNA to load β-clamp at nicks and small gaps
Source: bioRxiv. 2026 Jan 20:2026.01.17.700081. Preprint. [Version 2] doi: 10.64898/2026.01.17.700081 (PMC12871192; doi:10.64898/2026.01.17.700081)
Supplement: 1 [file NIHPP2026.01.17.700081V2-supplement-1.pdf]

bioRxiv preprint doi: https://doi.org/10.64898/2026.01.17.700081; this version posted January 20, 2026. The copyright holder for this preprint (which was not certified by peer review) is the author/funder, who has granted bioRxiv a license to display the preprint in perpetuity. It is made available under aCC-BY 4.0 International license.

Supplemental video legends

Video 1. Overview of thirteen β-clamp loading intermediates captured for DnaX-complex loading β-clamp onto either a 1-nt gapped DNA (five intermediates) or a 10-nt gapped DNA (eight intermediates). The thirteen atomic models are aligned and displayed individually, each rotated by 360º about the Y axis.

Video 2. Morphing of β-clamp loading onto a 1-nt gapped DNA substrate. The morph integrates previously reported structures of DnaX-complex loading β-clamp onto tailed DNA[16,17], with the five loading intermediates captured here using a 1-nt gapped DNA. The animation begins with formation of a binary DnaX-complex–β-clamp complex in the presence of ATP, which induces β-clamp gate opening. The open clamp then engages the 3'-dsDNA segment of the 1-nt gapped DNA via a conserved basic patch on the outer surface of the β-clamp near the open gate. This interaction is followed by sharp bending of the 5′-dsDNA towards the 3′-dsDNA, generating a stressed U-shaped DNA conformation and exposing the recessed 3′ end. The resulting DNA strain promotes insertion of the recessed 3′ end into the DnaX-complex chamber, entry of the 3′-dsDNA into the β-clamp, and binding of the 5′-dsDNA to the clamp basic patch vacated by the 3'-dsDNA. During this process, the δ-subunit Y316 sensor recognizes—but does not unwind—the recessed DNA 3′ end. Subsequent DNA engagement within the β-clamp chamber is proposed to drive clamp gate closure via electrostatic interactions, stimulate ATP hydrolysis, and trigger dissociation of the DnaX-complex, completing the clamp loading reaction.

bioRxiv preprint doi: https://doi.org/10.64898/2026.01.17.700081; this version posted January 20, 2026. The copyright holder for this preprint (which was not certified by peer review) is the author/funder, who has granted bioRxiv a license to display the preprint in perpetuity. It is made available under aCC-BY 4.0 International license.

Video 3. Morphing of β-clamp loading onto a larger gapped DNA (>6 nt). The animation was generated in ChimeraX based on the reported structures of the β-clamp (PDB: 1MMI)[87], the *E. coli* DnaX-complex bound to β-clamp in the absence of DNA (PDB: 8GIY and 7TI8)[16,17], and the eight loading intermediates captured here with a 10-nt gapped DNA. In the presence of ATP, the DnaX-complex binds and opens the β-clamp gate. Because the recessed DNA 3′ end is accessible in large-gapped substrates, it can directly enter the clamp loader chamber and engage the δ-subunit Y316 sensor residue, while the 3′-dsDNA binds to the basic patch on the exterior surface of β-clamp. Electrostatic attraction between the negatively charged DNA backbone and the positively charged interior of the β-clamp promotes insertion of the 3′-dsDNA into the clamp chamber and drives clamp gate closure, ultimately leading to dissociation of the DnaX-complex and formation of a DNA-encircling β-clamp.

bioRxiv preprint doi: https://doi.org/10.64898/2026.01.17.700081; this version posted January 20, 2026. The copyright holder for this preprint (which was not certified by peer review) is the author/funder, who has granted bioRxiv a license to display the preprint in perpetuity. It is made available under aCC-BY 4.0 International license.

Table S1. Cryo-EM data collection, refinement, and atomic model validation

**Structures**	**DnaX-complex–β-clamp–1-nt gapped DNA**					**DnaX-complex[a]**	**ψ-χ focus refined[a]**
	**State 1 conf. 1**	**State 1 conf. 2**	**State 1 conf. 3**	**State 2 conf. 1**	**State 2 conf. 2**		
EMDB ID	EMD-71013	EMD-71014	EMD-71015	EMD-71016	EMD-71017	EMD-71018	EMD-71020
PDB ID	9OYB	9OYC	9OYD	9OYE	9OYF	9OYG	
**Data collection**							
Magnification	105,000						
Voltage (kV)	300						
Dose (e^-/Å^2)	49.6					59	
Under-focus (μm)	1.2 - 1.6						
Pixel size (Å)	0.828						
Symmetry	C1						
Initial particle #	4.1 million					3.1 M	7.8 M
Final particle #	105,100	228,387	232,057	283,092	203,554	478,123	242,505
Map resolution (Å)	3.14	2.71	2.73	2.72	3.12	2.95	5.50
FSC threshold						0.143	
Map resolution range (Å)	2.7 – 11.0	2.4 – 11.0	2.3 – 12.0	2.4 – 10.3	2.7 – 10.1	2.5 – 9.5	4.5 – 13.0
**Refinement**							
Initial model used (PDB code)	1JR3, 3BEP					1JR3	3SXU
Map sharpening B factor (Å^2)	-105.2	-86.9	-87.1	-99.5	-107.2	-116.7	-211.4
Map to model CC_{mask}	0.89	0.89	0.88	0.88	0.88	0.79	
Model composition							
Non-hydrogen atoms	21,074	20,894	20,661	21,517	21,127	14,125	
Protein and DNA residues	2,532; 59	2,530; 51	2,529; 40	2,531; 81	2,531; 62	1794; 0	
Ligands	10	10	10	10	10	10	
R.m.s. deviations							
Bond lengths (Å)	0.004	0.004	0.003	0.006	0.005	0.003	
Bond angles (°)	0.756	0.631	0.594	0.684	0.669	0.623	
Validation							
MolProbity score	1.34	1.67	1.26	1.38	1.49	1.58	
Clashscore	5.53	4.56	4.99	6.08	5.47	8.42	
Poor rotamers (%)	0	0	0	0	0	0	
Ramachandran plot							
Favored (%)	97.81	98.33	98.29	97.77	96.86	97.35	
Allowed (%)	2.19	1.67	1.71	2.23	3.14	2.65	
Disallowed (%)	0	0	0	0	0	0	

[a]: The last two columns are from dataset with 10-nt gapped DNA (continued in **Table S2**).

bioRxiv preprint doi: https://doi.org/10.64898/2026.01.17.700081; this version posted January 20, 2026. The copyright holder for this preprint (which was not certified by peer review) is the author/funder, who has granted bioRxiv a license to display the preprint in perpetuity. It is made available under aCC-BY 4.0 International license.

Table S2. Cryo-EM data collection, refinement, and atomic model validation

	DnaX-complex–β-clamp–10-nt gapped DNA						
**Structures**	**DNA recognition**	**fully open, DNA unsettled**	**fully open, DNA settled**	**partially closed**	**fully closed 1**	**fully closed 2**	**fully closed 3**
EMDB ID	EMD-71021	EMD-71022	EMD-71023	EMD-71024	EMD-71025	EMD-71026	EMD-71027
PDB ID	9OYH	9OYI	9OYJ	9OYK	9OYL	9OYM	9OYN
**Data collection and processing**							
Magnification	105,000						
Voltage (kV)	300						
Electron dose ($e^-/Å^2$)	59						
Under-focus range (μm)	1.2 - 1.6						
Pixel size (Å)	0.828						
Symmetry imposed	C1						
Initial particle images (no.)	8,537,193						
Final particle images (no.)	473,295	502,506	499,551	476,182	360,948	539,340	443,597
Map resolution (Å)	2.54	2.54	2.53	2.60	2.88	2.60	2.70
FSC threshold	0.143						
Map resolution range (Å)	2.2 – 9.5	2.2 – 9.3	2.2 – 9.5	2.3 – 9.6	1.8 – 9.9	2.3 – 9.4	2.4 – 9.9
**Refinement**							
Initial model used (PDB code)	1JR3, 3BEP, 3SXU						
Map sharpening B factor ($Å^2$)	-91.6	-92.5	-92.6	-95.7	-99.1	-98.1	-99.0
Map to model CC_{mask}	0.71	0.87	0.75	0.87	0.75	0.86	0.82
Model composition							
Non-hydrogen atoms	22,702	20,796	20,835	20,861	20,835	20,861	20,843
Protein and DNA residues	2,779; 44	2,531; 46	2,531; 47	2,534; 47	2,532; 47	2,534; 47	2532; 47
Ligands	10	10	11	11	11	11	11
R.m.s. deviations							
Bond lengths (Å)	0.002	0.005	0.007	0.003	0.003	0.004	0.005
Bond angels (º)	0.480	0.634	0.687	0.530	0.489	0.587	0.579
Validation							
MolProbity score	1.40	1.21	1.52	1.24	1.45	1.21	1.37
Clashscore	7.29	4.31	6.67	4.73	6.98	4.25	5.19

bioRxiv preprint doi: https://doi.org/10.64898/2026.01.17.700081; this version posted January 20, 2026. The copyright holder for this preprint (which was not certified by peer review) is the author/funder, who has granted bioRxiv a license to display the preprint in perpetuity. It is made available under aCC-BY 4.0 International license.

Poor rotamers (%)	0	0	0	0	0	0	0
Ramachandran plot							
Favored (%)	98.15	98.17	97.13	98.13	97.69	98.57	97.54
Allowed (%)	1.85	1.83	2.87	1.87	2.31	1.43	2.46
Disallowed (%)	0	0	0	0	0	0	0

bioRxiv preprint doi: https://doi.org/10.64898/2026.01.17.700081; this version posted January 20, 2026. The copyright holder for this preprint (which was not certified by peer review) is the author/funder, who has granted bioRxiv a license to display the preprint in perpetuity. It is made available under aCC-BY 4.0 International license.

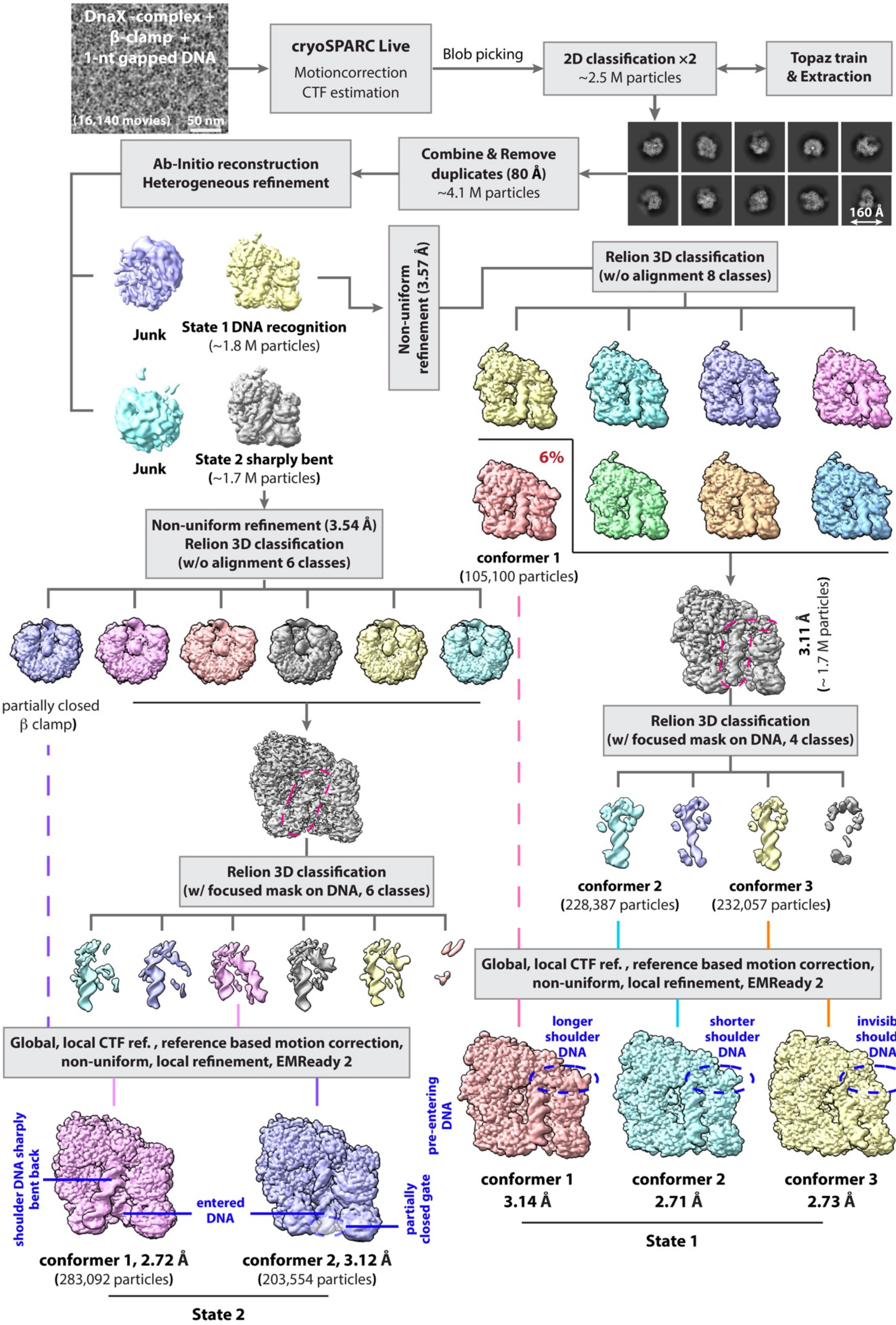

Figure S1. Cryo-EM data processing of the DnaX-complex–β-clamp bound to 1-nt gapped DNA. A representative micrograph from approximately 16,000 movies collected on a 300 kV Titan Krios

bioRxiv preprint doi: https://doi.org/10.64898/2026.01.17.700081; this version posted January 20, 2026. The copyright holder for this preprint (which was not certified by peer review) is the author/funder, who has granted bioRxiv a license to display the preprint in perpetuity. It is made available under aCC-BY 4.0 International license.

microscope is shown, with scale bar indicated. Image processing was performed using cryoSPARC v4.5 and Relion v5.0[68,88]. Two major clamp loading states were identified: a first state comprising three conformers and a second state comprising two conformers.

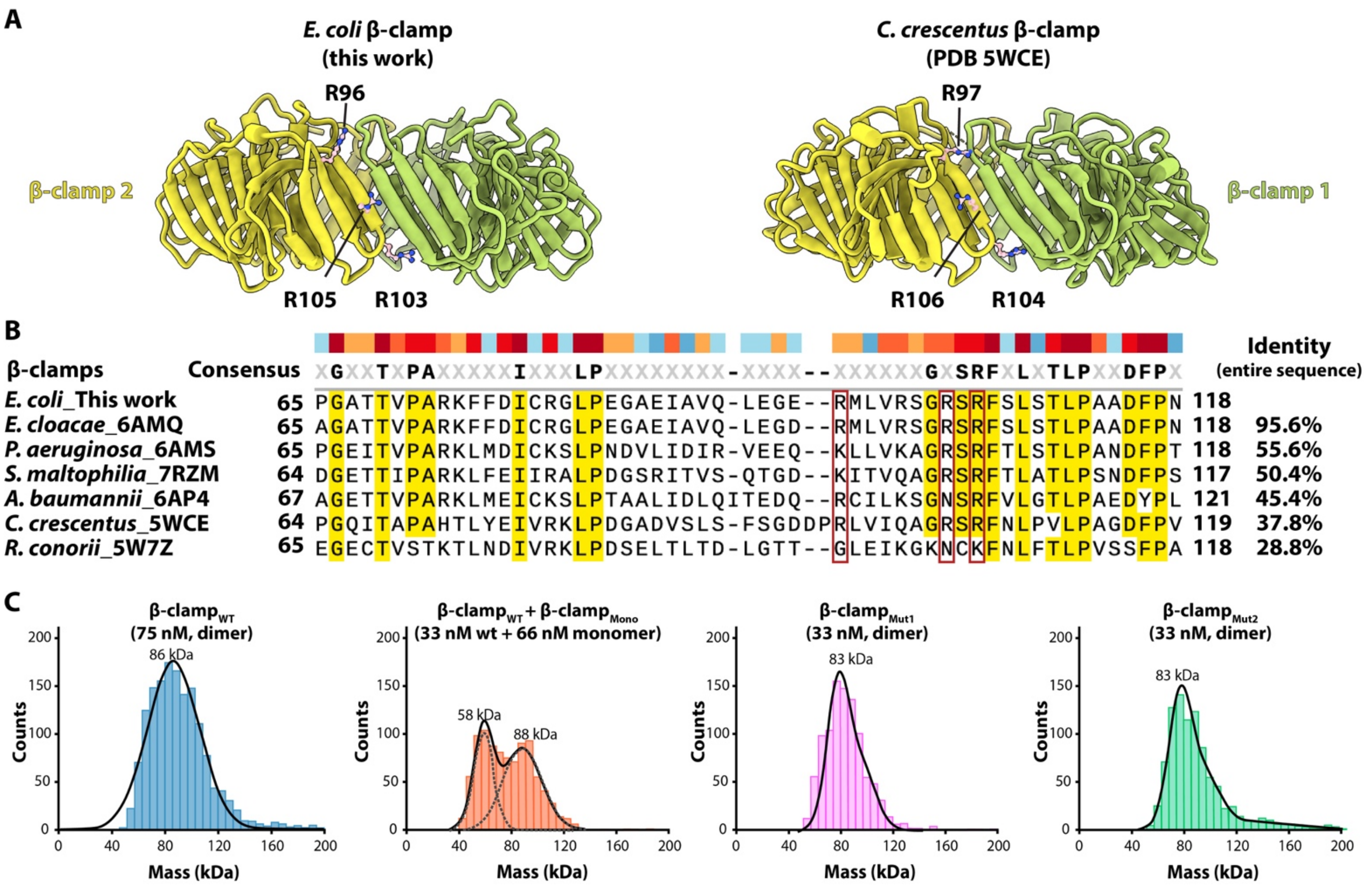

Figure S2. The basic patch on the outer surface of the β-clamp is conserved among prokaryotes. Related to Figure 3. (**A**) Structural comparison of the *E. coli* β-clamp with that of *Caulobacter crescentus* as a representative example. The three positively charged residues that form the conserved basic patch are shown as sticks and are located on the outer surface near the left side of the DNA entry gate between the two β subunits. (**B**) Structure-based sequence alignment of the β-clamp region containing the three basic residues, highlighted by red boxes. Structures were aligned using ChimeraX[86], and PDB accession code for each structure is listed following the species name. (**C**) The β-clamp mutants used in this study remain dimeric in solution. Mass photometry measurements have a low molecular weight detection limit of ~40 kDa; therefore, apparent β monomer mass values are expected to be slightly higher than theoretical monomer mass. As controls, wild-type β-clamp dimers and a previously characterized β monomer mutant, in which the hydrophobic core residues were mutated to disrupt dimerization (I272A/L273/A)[89] were analyzed. Both β-clamp$_{Mut1}$ and β-clamp$_{Mut2}$, harboring R96, R103, and R105 substitutions to alanine or glutamate, respectively, remained dimeric in solution.

bioRxiv preprint doi: https://doi.org/10.64898/2026.01.17.700081; this version posted January 20, 2026. The copyright holder for this preprint (which was not certified by peer review) is the author/funder, who has granted bioRxiv a license to display the preprint in perpetuity. It is made available under aCC-BY 4.0 International license.

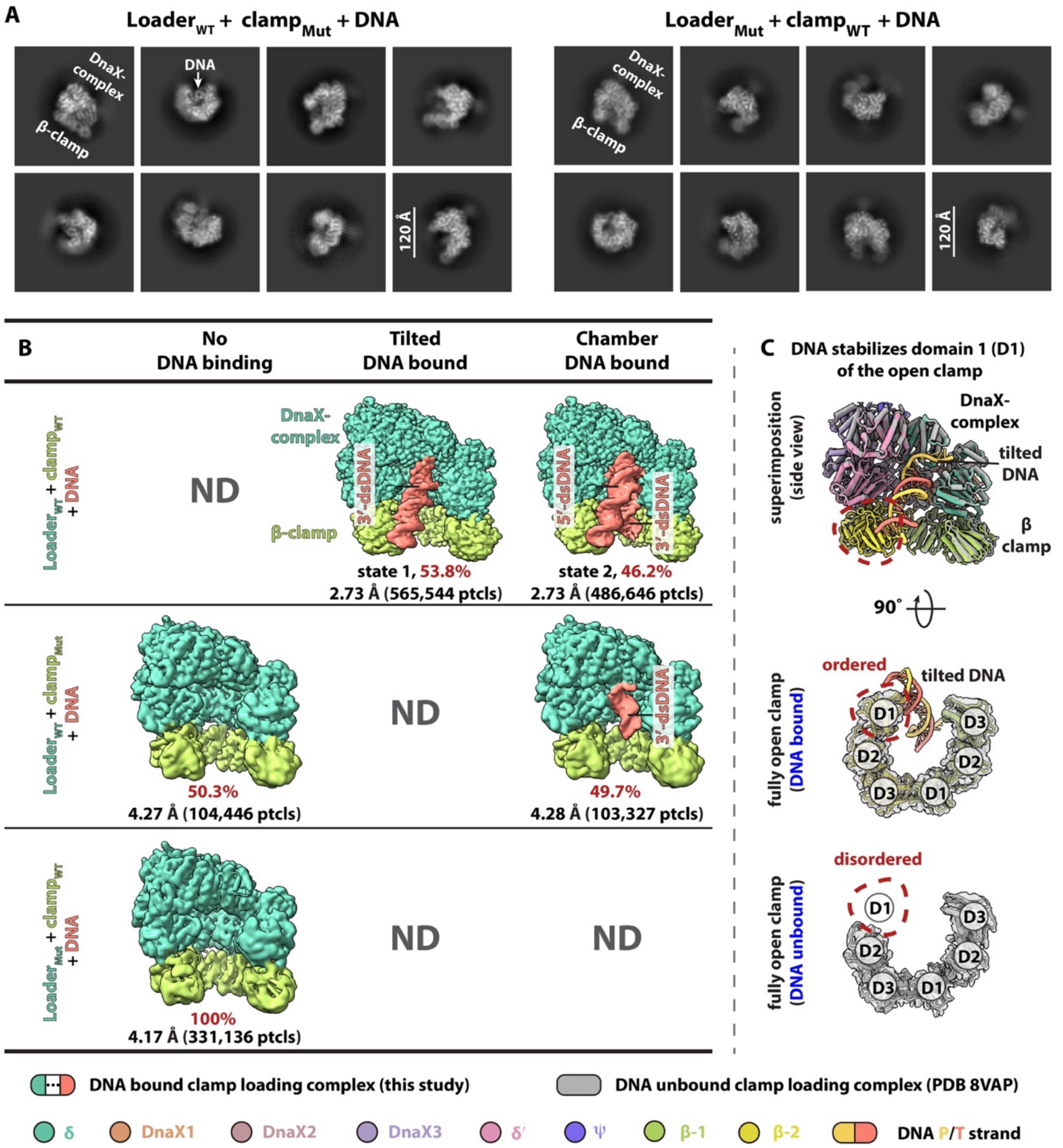

Figure S3. Cryo-EM analysis of the effects of mutations on β-clamp loading. (**A**) Representative 2D class averages of assembled clamp loading complexes containing mutant components. Left: wild-type DnaX-complex (loader$_{WT}$) assembled with a β-clamp basic patch mutant harboring R96A, R103A, and R105A substitutions (β-clamp$_{Mut}$) and 1-nt gapped DNA. Right: DnaX-complex bearing the δ-subunit Y316A substitution (loader$_{Mut}$) assembled with wild-type β-clamp (clamp$_{WT}$) and 1-nt gapped DNA. Assemblies were performed under same conditions as used for wild-type complexes. Key components and scale bar are indicated. (**B**) Reconstructed cryo-EM maps corresponding to the assemblies shown in (A). For comparison, representative maps from wild-type clamp and clamp loader assemblies are also shown (state 1 conformer 1 and state 2 conformer 1). Total particle numbers for each state (summed across conformers) are listed to indicate relative populations. For assemblies containing wild-type clamp and loader, no clamp–clamp loader–only complex (lacking DNA) was observed. In contrast, assemblies

bioRxiv preprint doi: https://doi.org/10.64898/2026.01.17.700081; this version posted January 20, 2026. The copyright holder for this preprint (which was not certified by peer review) is the author/funder, who has granted bioRxiv a license to display the preprint in perpetuity. It is made available under aCC-BY 4.0 International license.

containing β-clamp$_{Mut}$ yielded approximately equal populations of clamp–clamp loader–only complexes and complexes containing chamber-bound 3′-dsDNA, but no intermediates with tilted 3′-dsDNA or bent 5′-were detected, consistent with the requirement for the β-clamp basic patch in DNA binding. For assemblies containing loader$_{Mut}$, only clamp–clamp loader–only complex was observed, with no DNA - bound state observed, indicating that the δ-subunit Y316 is critical for DNA recognition and subsequent clamp loading. ND, not detected. (**C**) DNA binding at the β-clamp basic patch stabilizes β-clamp domain I. Superimposition of structures of the DnaX-complex with an open β-clamp in the absence of DNA (gray, PDB: 8VAP)[17] and in the presence of 1-nt gapped DNA (colored; state 1 conformer 1 from this study) is showed. The clamp loader is omitted in the bottom views for clarity.

bioRxiv preprint doi: https://doi.org/10.64898/2026.01.17.700081; this version posted January 20, 2026. The copyright holder for this preprint (which was not certified by peer review) is the author/funder, who has granted bioRxiv a license to display the preprint in perpetuity. It is made available under aCC-BY 4.0 International license.

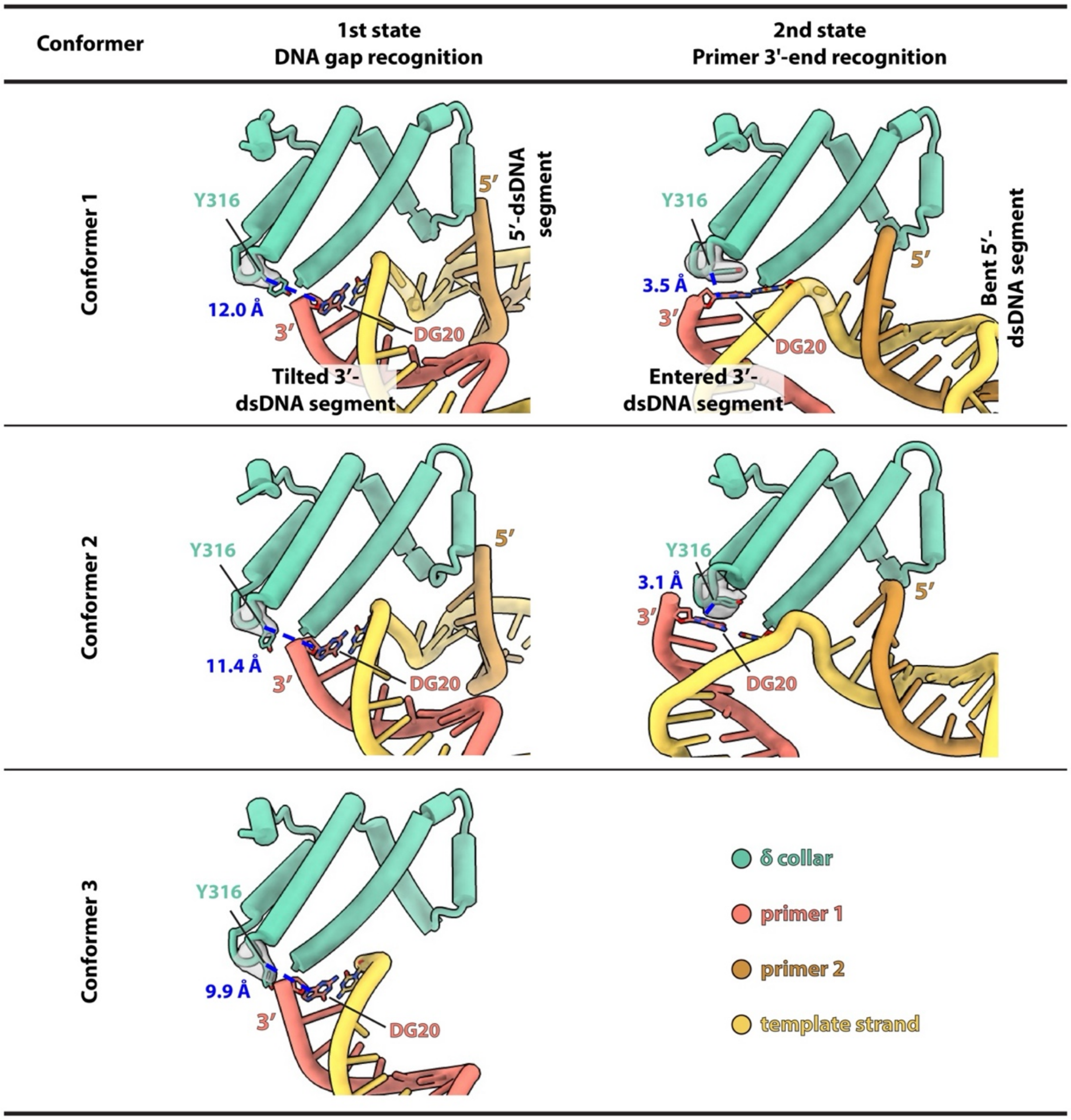

Figure S4. The *E. coli* clamp loader residue δ Y316 recognizes the recessed DNA 3′ end to facilitate proper positioning of the 3′-dsDNA within the loader chamber. In the first DNA gap recognition state, comparison of conformers 1–3 shows a progressive decrease in the distance between δ Y316 and the DNA 3′ end, from 12.0 Å to 9.9 Å, accompanied by increasingly well-defined local cryo-EM density for Y316. In the second state, corresponding to DNA primer 3′ end recognition, this distance is further reduced to ~3.3 Å in both conformers, and Y316 adopts a base-mimicking conformation that stacks against the terminal base G20 of the primer 3′ end, resulting in full stabilization of this residue. For clarity, only a closed-up view of this region is shown. Distance were measured between the Y316 Cβ atom and G20 C8 atom, which approximates the distance between the centers of the two ring-containing residues in the second state.

bioRxiv preprint doi: https://doi.org/10.64898/2026.01.17.700081; this version posted January 20, 2026. The copyright holder for this preprint (which was not certified by peer review) is the author/funder, who has granted bioRxiv a license to display the preprint in perpetuity. It is made available under aCC-BY 4.0 International license.

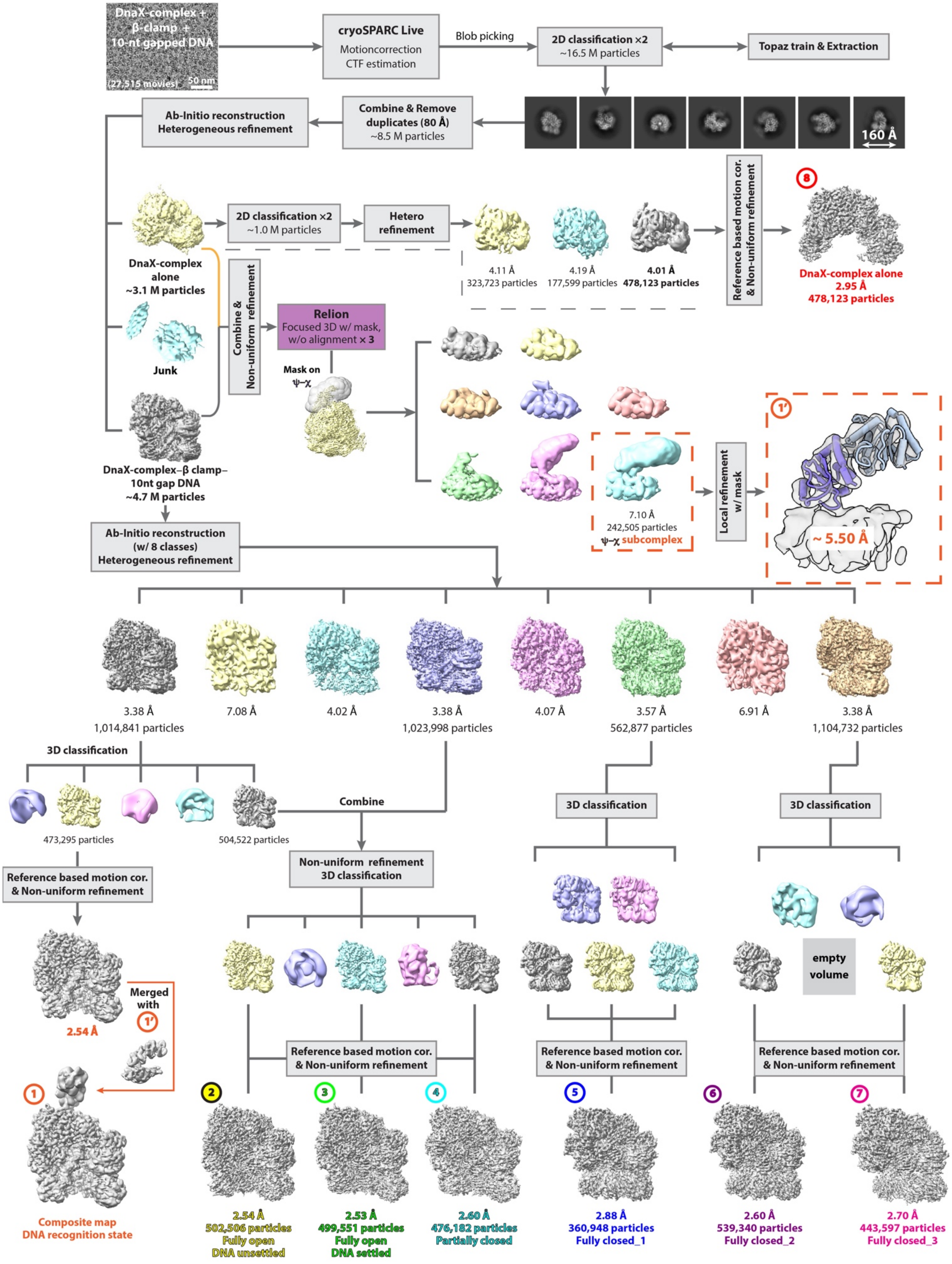

bioRxiv preprint doi: https://doi.org/10.64898/2026.01.17.700081; this version posted January 20, 2026. The copyright holder for this preprint (which was not certified by peer review) is the author/funder, who has granted bioRxiv a license to display the preprint in perpetuity. It is made available under aCC-BY 4.0 International license.

Figure S5. Cryo-EM data processing of the DnaX-complex–β-clamp bound to 10-nt gapped DNA. A representative micrograph from ~28,000 movies collected on a 300kV Titan Krios microscope is shown, with scale bar indicated. Image processing was performed using cryoSPARC v4.5 and Relion v5.0[68,88]. Eight distinct clamp loading states were resolved at resolutions from ~3.0 Å to 2.5 Å, with density corresponding to the χψ heterodimer merged into the state 2 map. The χψ crystal structure bound to an SSB peptide bound (PDB: 3SXU) was rigid-body fitted into the locally refined 3D map highlighted by dashed red squares.

bioRxiv preprint doi: https://doi.org/10.64898/2026.01.17.700081; this version posted January 20, 2026. The copyright holder for this preprint (which was not certified by peer review) is the author/funder, who has granted bioRxiv a license to display the preprint in perpetuity. It is made available under aCC-BY 4.0 International license.

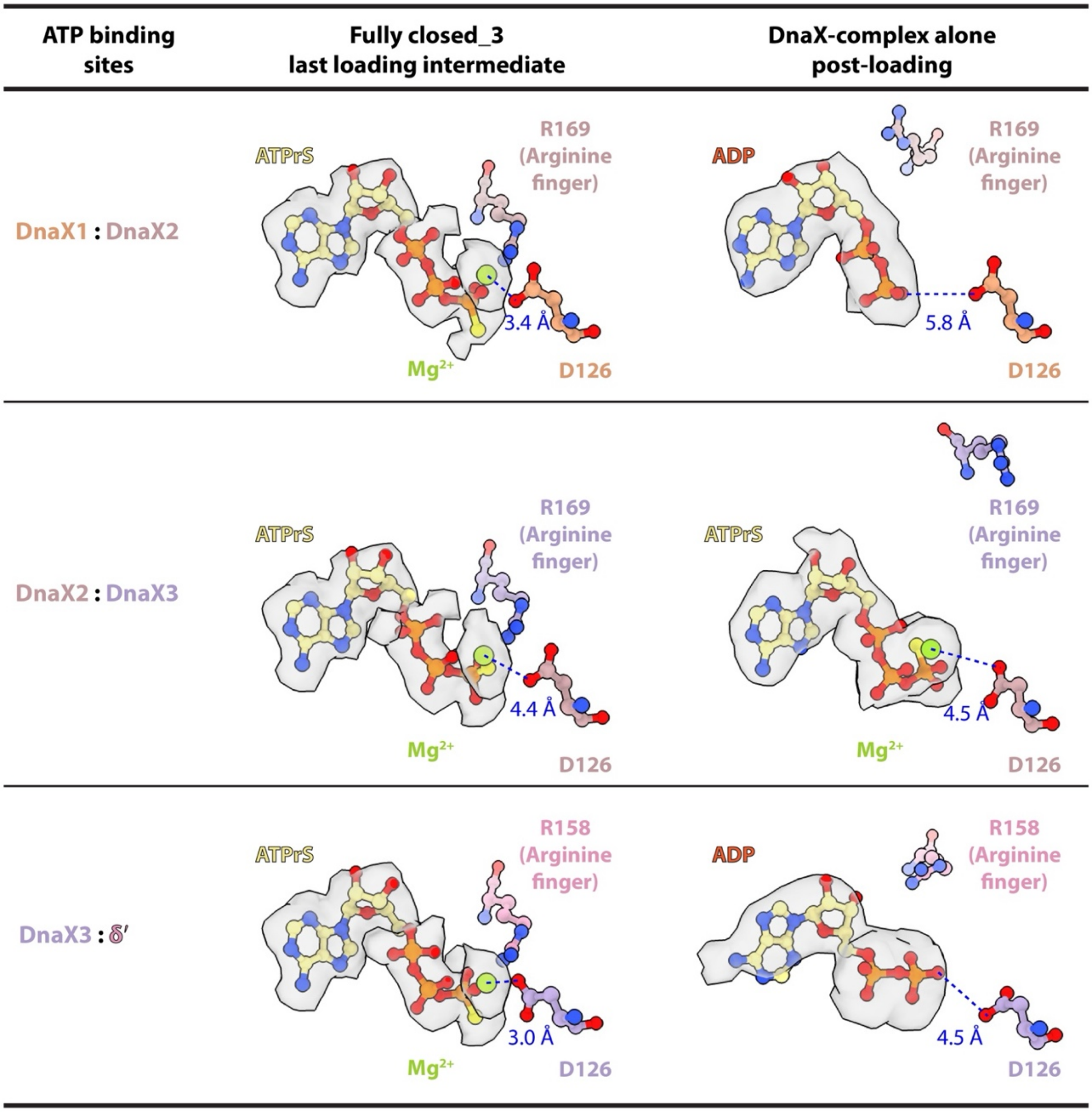

Figure S6. ATPγS hydrolysis is observed only in the DnaX-complex–alone structure. Across all 13 clamp loading intermediates captured with either 1-nt or 10-nt gapped DNA, ATPγS was bound to all ATPase sites from the fully open β-clamp state through the final fully closed state, indicating no ATPγS hydrolysis during these intermediates. The last loading intermediates (fully closed_3) is shown here as a representative example. In this state, the distances between the Mg^{2+} ion and the catalytic residue D126 (Walker B/DEAD box) are 3.4 Å and 3.0 Å at the first and third ATPase sites, respectively, consistent with a geometry competent for hydrolysis, whereas the distance at the second site is 4.4 Å. By contrast, in the DnaX-complex–alone structure, ADP can be unambiguously modeled at the first and third sites, while ATPγS remained bound at the second site, indicating that ATPγS hydrolysis had occurred at two sites following clamp loading. For clarity, only the bound nucleotides and key catalytic residues are shown.

bioRxiv preprint doi: https://doi.org/10.64898/2026.01.17.700081; this version posted January 20, 2026. The copyright holder for this preprint (which was not certified by peer review) is the author/funder, who has granted bioRxiv a license to display the preprint in perpetuity. It is made available under aCC-BY 4.0 International license.

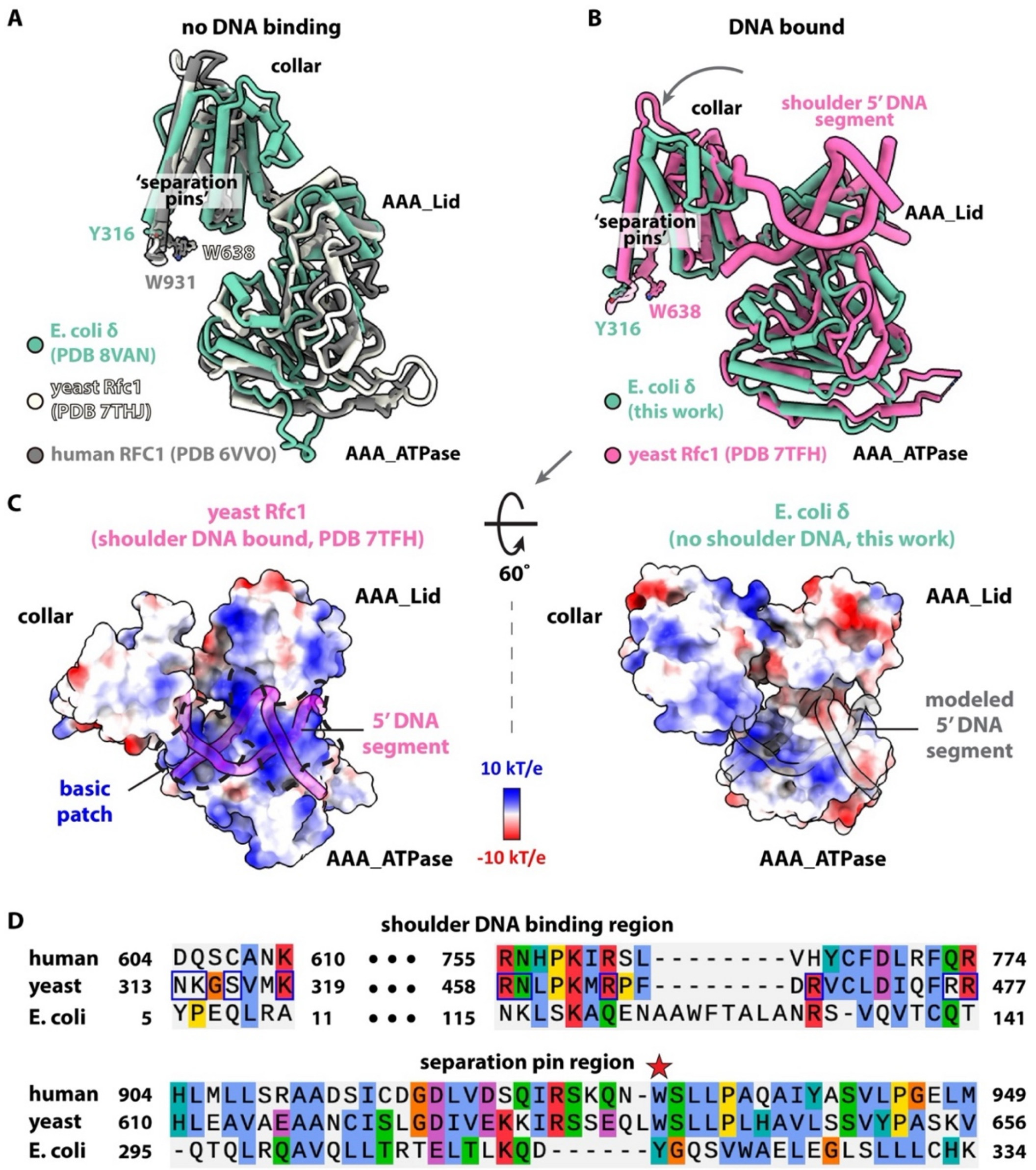

Figure S7. Comparisons of the clamp loader first subunit from *E. coli*, yeast, and human. (**A**) In the absence of DNA binding, the overall structures of *E. coli* δ, yeast Rfc1, and human RFC1 are similar. (**B**) Upon binding of the 3′-dsDNA in the chamber (omitted), the yeast Rfc1 AAA Lid and Collar domains undergo a larger movement than those in *E. coli*, creating additional space to facilitate subsequent binding of 5′-dsDNA at the shoulder. (**C**) The Rfc1 shoulder region presents a large basic patch (black dashed line) for 5′-dsDNA binding (left), which is absent in *E. coli* δ; steric clashes are observed when the Rfc1-bound 5′-dsDNA is modeled onto *E. coli* δ (right). (**D**) Structural-based sequence alignment shows that the shoulder regions of yeast Rfc1 and human RFC1 are more positively charged than that of *E. coli* δ (upper). The 'separation pin' in human and yeast proteins is a Trp residue with two aromatic rings, whereas the *E. coli* protein contains a Tyr residue with a single aromatic ring (lower). Residues are colored by chemical property (scheme from the MUSCLE alignment program[90]).

bioRxiv preprint doi: https://doi.org/10.64898/2026.01.17.700081; this version posted January 20, 2026. The copyright holder for this preprint (which was not certified by peer review) is the author/funder, who has granted bioRxiv a license to display the preprint in perpetuity. It is made available under aCC-BY 4.0 International license.

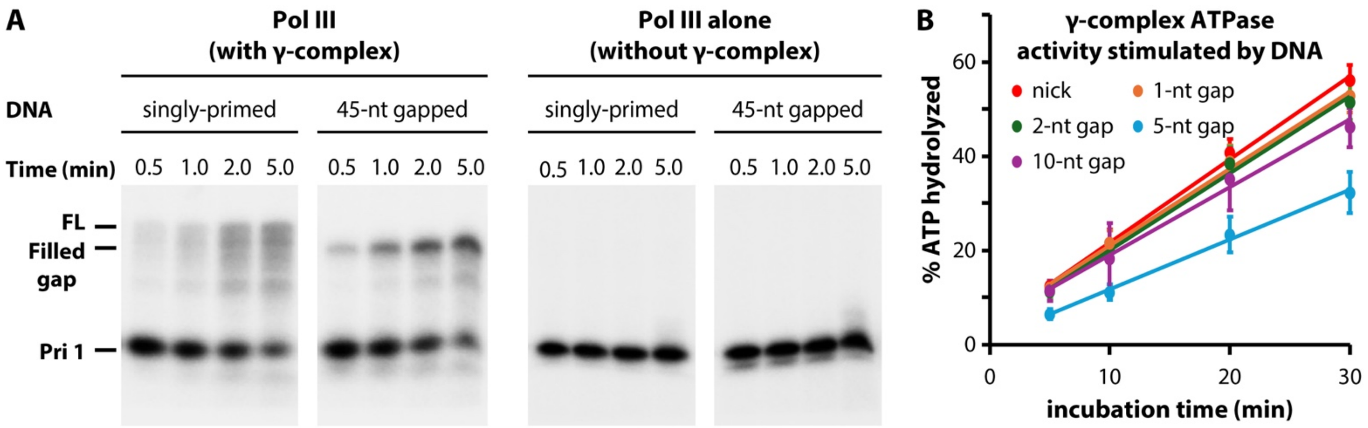

Figure S8. Characterization of Pol III DNA synthesis and DnaX-complex ATPase activity on distinct DNA substrates. (**A**) Urea–PAGE analysis of ^{32}P-labelled primer extension over increasing loading times using either singly primed or gapped DNA, in the presence (left) or absence (right) of the γ-complex. A sufficient amount of Pol III was added to saturate the reaction, making clamp loading the rate limiting step. (**B**) Stimulation of DnaX-complex ATPase activities by DNA substrates containing gaps of different sizes. See **Methods** for details.

bioRxiv preprint doi: https://doi.org/10.64898/2026.01.17.700081; this version posted January 20, 2026. The copyright holder for this preprint (which was not certified by peer review) is the author/funder, who has granted bioRxiv a license to display the preprint in perpetuity. It is made available under aCC-BY 4.0 International license.

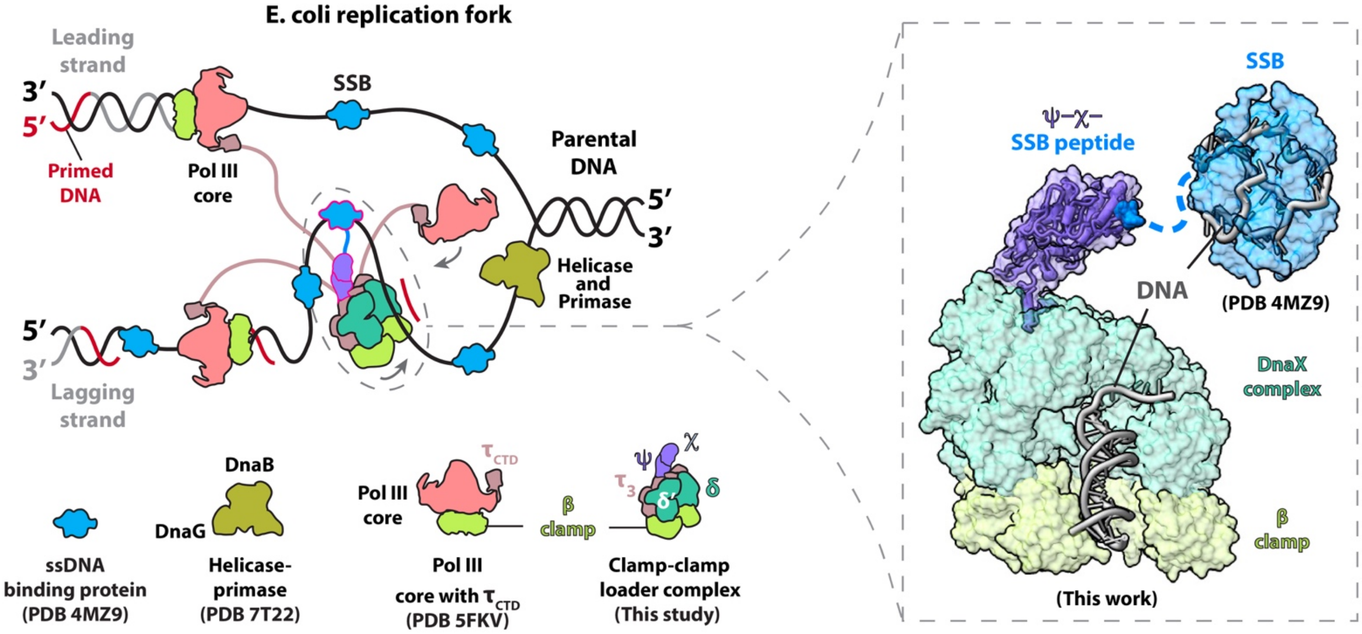

Figure S9. Model for coordination of the replisome by the DnaX-holo-complex. Left, a simplified model of the *E. coli* replication fork is shown, including the DnaB helicase encircling the lagging strand and unwinding parental DNA, the DnaG primase bound to DnaB and synthesizing RNA primers on the lagging strand template, and multiple SSB molecules coating the ssDNA. In addition to loading the β-clamp for Pol III on both the leading and lagging strands, the DnaX-holo-complex organizes the replication fork by interacting with SSB via its χ subunit and with three Pol III molecules via the C-terminal domains of its three τ subunits (τ_{CTD})[61,62]. These interactions may facilitate recruitment of Pol III to the loaded β-clamp. Key components are listed together with the available PDB codes. Right, a structural model shows SSB bound to ssDNA and interacting with the DnaX-holo-complex through its flexibly linked C-terminal peptide (blue surface).
